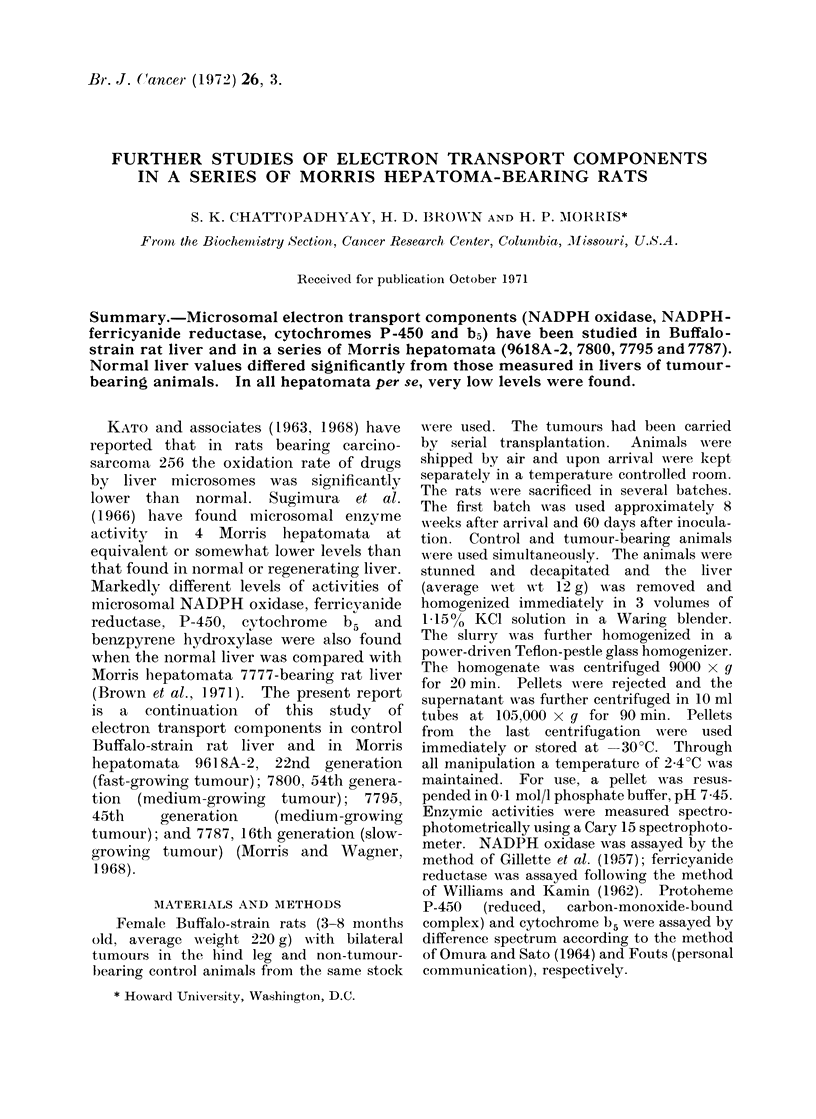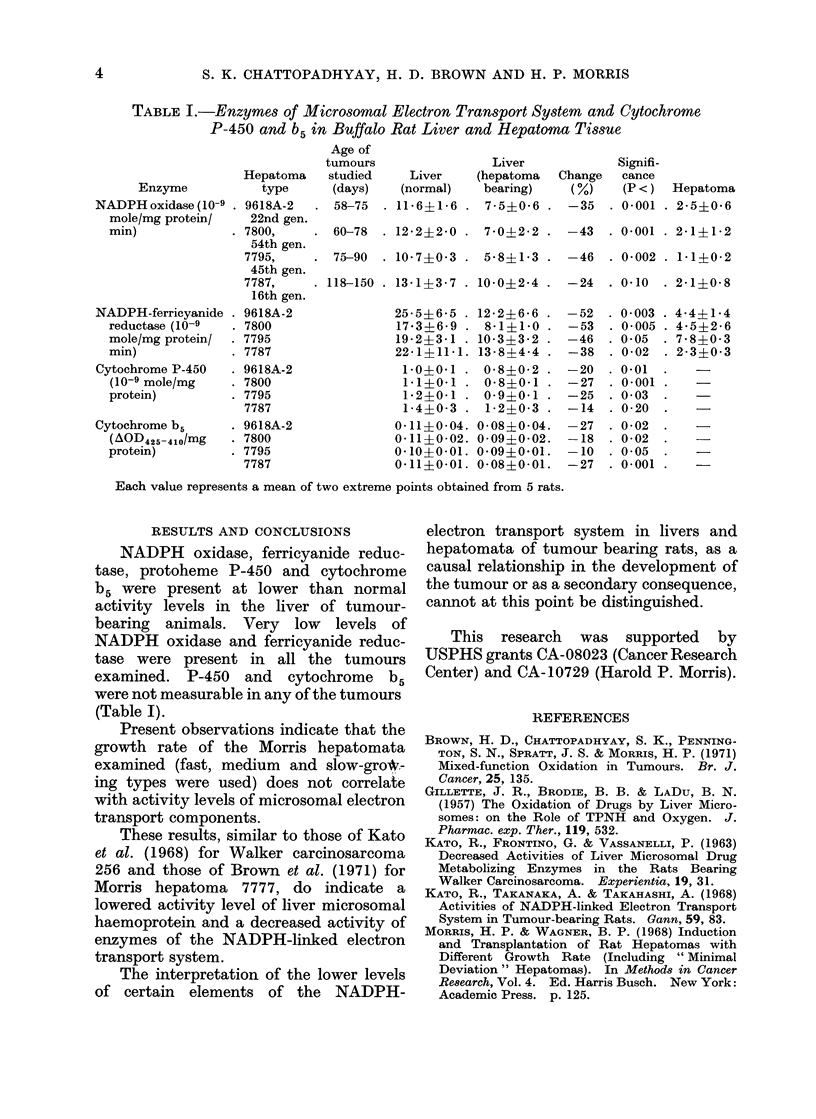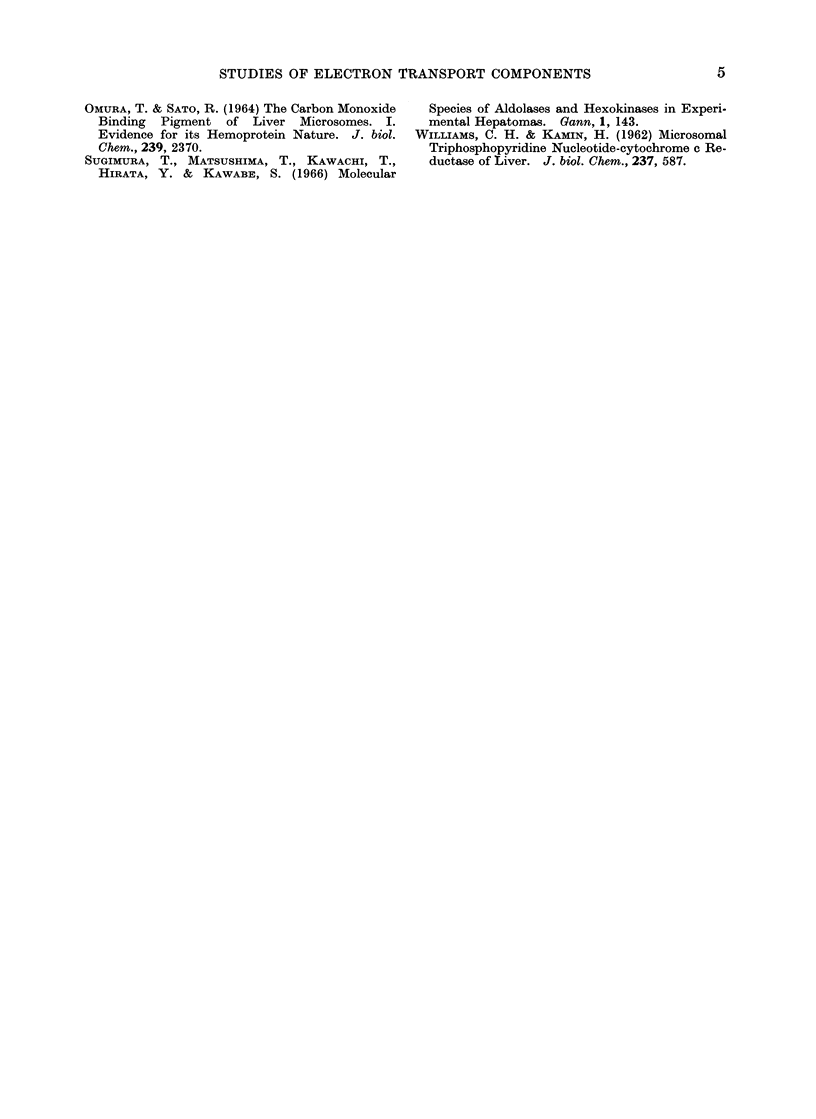# Further Studies of Electron Transport Components in a Series of Morris Hepatoma-Bearing Rats

**DOI:** 10.1038/bjc.1972.2

**Published:** 1972-02

**Authors:** S. K. Chattopadhyay, H. D. Brown, H. P. Morris

## Abstract

Microsomal electron transport components (NADPH oxidase, NADPH-ferricyanide reductase, cytochromes P-450 and b_5_) have been studied in Buffalostrain rat liver and in a series of Morris hepatomata (9618A-2, 7800, 7795 and 7787). Normal liver values differed significantly from those measured in livers of tumourbearing animals. In all hepatomata *per se,* very low levels were found.


*Br. J. Cancer* (1972) **26**, 3.

### FURTHER STUDIES OF ELECTRON TRANSPORT COMPONENTS IN A SERIES OF MORRIS HEPATOMA-BEARING RATS

S. K. CHATTOPADHYAY, H. D. BROWN AND H. P. MORRIS*

*From the Biochemistry Section, Cancer Research Center, Columbia, Missouri, U.S.A.*

Received for publication October 1971

**Summary.—Microsomal electron transport components (NADPH oxidase, NADPH-ferricyanide reductase, cytochromes P-450 and $b_5$) have been studied in Buffalo-strain rat liver and in a series of Morris hepatomata (9618A-2, 7800, 7795 and 7787). Normal liver values differed significantly from those measured in livers of tumour-bearing animals. In all hepatomata *per se*, very low levels were found.**

KATO and associates (1963, 1968) have reported that in rats bearing carcinosarcoma 256 the oxidation rate of drugs by liver microsomes was significantly lower than normal. Sugimura *et al.* (1966) have found microsomal enzyme activity in 4 Morris hepatomata at equivalent or somewhat lower levels than that found in normal or regenerating liver. Markedly different levels of activities of microsomal NADPH oxidase, ferricyanide reductase, P-450, cytochrome $b_5$ and benzpyrene hydroxylase were also found when the normal liver was compared with Morris hepatomata 7777-bearing rat liver (Brown *et al.*, 1971). The present report is a continuation of this study of electron transport components in control Buffalo-strain rat liver and in Morris hepatomata 9618A-2, 22nd generation (fast-growing tumour); 7800, 54th generation (medium-growing tumour); 7795, 45th generation (medium-growing tumour); and 7787, 16th generation (slow-growing tumour) (Morris and Wagner, 1968).

### MATERIALS AND METHODS

Female Buffalo-strain rats (3–8 months old, average weight 220 g) with bilateral tumours in the hind leg and non-tumour-bearing control animals from the same stock were used. The tumours had been carried by serial transplantation. Animals were shipped by air and upon arrival were kept separately in a temperature controlled room. The rats were sacrificed in several batches. The first batch was used approximately 8 weeks after arrival and 60 days after inoculation. Control and tumour-bearing animals were used simultaneously. The animals were stunned and decapitated and the liver (average wet wt 12 g) was removed and homogenized immediately in 3 volumes of 1·15% KCl solution in a Waring blender. The slurry was further homogenized in a power-driven Teflon-pestle glass homogenizer. The homogenate was centrifuged $9000 \times g$ for 20 min. Pellets were rejected and the supernatant was further centrifuged in 10 ml tubes at $105{,}000 \times g$ for 90 min. Pellets from the last centrifugation were used immediately or stored at −30°C. Through all manipulation a temperature of 2·4°C was maintained. For use, a pellet was resuspended in 0·1 mol/l phosphate buffer, pH 7·45. Enzymic activities were measured spectrophotometrically using a Cary 15 spectrophotometer. NADPH oxidase was assayed by the method of Gillette *et al.* (1957); ferricyanide reductase was assayed following the method of Williams and Kamin (1962). Protoheme P-450 (reduced, carbon-monoxide-bound complex) and cytochrome $b_5$ were assayed by difference spectrum according to the method of Omura and Sato (1964) and Fouts (personal communication), respectively.

* Howard University, Washington, D.C.

TABLE I.—*Enzymes of Microsomal Electron Transport System and Cytochrome P-450 and $b_5$ in Buffalo Rat Liver and Hepatoma Tissue*

| Enzyme | Hepatoma type | Age of tumours studied (days) | Liver (normal) | Liver (hepatoma bearing) | Change (%) | Significance (P < ) | Hepatoma |
|---|---|---|---|---|---|---|---|
| NADPH oxidase ($10^{-9}$ mole/mg protein/ min) | 9618A-2 22nd gen. | 58–75 | 11·6±1·6 | 7·5±0·6 | −35 | 0·001 | 2·5±0·6 |
| | 7800, 54th gen. | 60–78 | 12·2±2·0 | 7·0±2·2 | −43 | 0·001 | 2·1±1·2 |
| | 7795, 45th gen. | 75–90 | 10·7±0·3 | 5·8±1·3 | −46 | 0·002 | 1·1±0·2 |
| | 7787, 16th gen. | 118–150 | 13·1±3·7 | 10·0±2·4 | −24 | 0·10 | 2·1±0·8 |
| NADPH-ferricyanide reductase ($10^{-9}$ mole/mg protein/ min) | 9618A-2 | | 25·5±6·5 | 12·2±6·6 | −52 | 0·003 | 4·4±1·4 |
| | 7800 | | 17·3±6·9 | 8·1±1·0 | −53 | 0·005 | 4·5±2·6 |
| | 7795 | | 19·2±3·1 | 10·3±3·2 | −46 | 0·05 | 7·8±0·3 |
| | 7787 | | 22·1±11·1 | 13·8±4·4 | −38 | 0·02 | 2·3±0·3 |
| Cytochrome P-450 ($10^{-9}$ mole/mg protein) | 9618A-2 | | 1·0±0·1 | 0·8±0·2 | −20 | 0·01 | — |
| | 7800 | | 1·1±0·1 | 0·8±0·1 | −27 | 0·001 | — |
| | 7795 | | 1·2±0·1 | 0·9±0·1 | −25 | 0·03 | — |
| | 7787 | | 1·4±0·3 | 1·2±0·3 | −14 | 0·20 | — |
| Cytochrome $b_5$ ($\Delta OD_{425-410}$/mg protein) | 9618A-2 | | 0·11±0·04 | 0·08±0·04 | −27 | 0·02 | — |
| | 7800 | | 0·11±0·02 | 0·09±0·02 | −18 | 0·02 | — |
| | 7795 | | 0·10±0·01 | 0·09±0·01 | −10 | 0·05 | — |
| | 7787 | | 0·11±0·01 | 0·08±0·01 | −27 | 0·001 | — |

Each value represents a mean of two extreme points obtained from 5 rats.

### RESULTS AND CONCLUSIONS

NADPH oxidase, ferricyanide reductase, protoheme P-450 and cytochrome $b_5$ were present at lower than normal activity levels in the liver of tumour-bearing animals. Very low levels of NADPH oxidase and ferricyanide reductase were present in all the tumours examined. P-450 and cytochrome $b_5$ were not measurable in any of the tumours (Table I).

Present observations indicate that the growth rate of the Morris hepatomata examined (fast, medium and slow-growing types were used) does not correlate with activity levels of microsomal electron transport components.

These results, similar to those of Kato *et al.* (1968) for Walker carcinosarcoma 256 and those of Brown *et al.* (1971) for Morris hepatoma 7777, do indicate a lowered activity level of liver microsomal haemoprotein and a decreased activity of enzymes of the NADPH-linked electron transport system.

The interpretation of the lower levels of certain elements of the NADPH-electron transport system in livers and hepatomata of tumour bearing rats, as a causal relationship in the development of the tumour or as a secondary consequence, cannot at this point be distinguished.

This research was supported by USPHS grants CA-08023 (Cancer Research Center) and CA-10729 (Harold P. Morris).

### REFERENCES

BROWN, H. D., CHATTOPADHYAY, S. K., PENNINGTON, S. N., SPRATT, J. S. & MORRIS, H. P. (1971) Mixed-function Oxidation in Tumours. *Br. J. Cancer*, **25**, 135.

GILLETTE, J. R., BRODIE, B. B. & LADU, B. N. (1957) The Oxidation of Drugs by Liver Microsomes: on the Role of TPNH and Oxygen. *J. Pharmac. exp. Ther.*, **119**, 532.

KATO, R., FRONTINO, G. & VASSANELLI, P. (1963) Decreased Activities of Liver Microsomal Drug Metabolizing Enzymes in the Rats Bearing Walker Carcinosarcoma. *Experientia*, **19**, 31.

KATO, R., TAKANAKA, A. & TAKAHASHI, A. (1968) Activities of NADPH-linked Electron Transport System in Tumour-bearing Rats. *Gann*, **59**, 83.

MORRIS, H. P. & WAGNER, B. P. (1968) Induction and Transplantation of Rat Hepatomas with Different Growth Rate (Including "Minimal Deviation" Hepatomas). In *Methods in Cancer Research*, Vol. 4. Ed. Harris Busch. New York: Academic Press. p. 125.

OMURA, T. & SATO, R. (1964) The Carbon Monoxide Binding Pigment of Liver Microsomes. I. Evidence for its Hemoprotein Nature. *J. biol. Chem.*, **239**, 2370.

SUGIMURA, T., MATSUSHIMA, T., KAWACHI, T., HIRATA, Y. & KAWABE, S. (1966) Molecular Species of Aldolases and Hexokinases in Experimental Hepatomas. *Gann*, **1**, 143.

WILLIAMS, C. H. & KAMIN, H. (1962) Microsomal Triphosphopyridine Nucleotide-cytochrome c Reductase of Liver. *J. biol. Chem.*, **237**, 587.